# Our New York Letter

**Published:** 1890-03

**Authors:** 


### Correspondence.

### OUR NEW YORK LETTER.

NEW YORK, February 13, 1890.

The topic of greatest general interest in medical circles here during the past month has been the epidemic. During one week the usual death rate for this season of the year was doubled. Funerals had been postponed as undertakers were unable to keep their engagements. Dispensaries and hospitals were crowded, many physicians were sick and all were busy. All this is practically ended now, but traces of "La Grippe" are still to be found.

Although the general opinion is that the late epidemic was one of influenza, differing in no essential from previous epidemics of that disease which have swept over Europe and America about once in ten years; yet a few claim that it corresponded more closely to dengue or break-bone fever, epidemics of which have, I believe, visited several of the Southern States within recent years.

The first news of it came in October, from one of the most easterly provinces of Russia. Early in November it had reached St. Petersburg, and by the end of that month Berlin. The first week in December found it in Paris, Southern Germany and Austria. It arrived in London with the New Year, and about a week later we had it here. Since then it has traveled westward and has prevailed extensively throughout the western and north-western States and Canada.

The symptoms in the majority of cases, were a chill followed by fever, headache, giddiness, pains in the loins and limbs, muscular weakness, and mental hebitude, varying grades of coryza, pharyngitis, and bronchial catarrh. The fever and many of the acuter symptoms subsided under treatment in from twenty-four hours to two or three days, and were followed by protracted muscular prostration and mental depression, with indefinite neuralgic pains, poor appetite, rapid pulse and sweating, ending in a slow recovery.

Such was the history of the mass of cases. Exceptionally other symptoms were present, or one or more of those enumerated became so pronounced as to overshadow the others. In a small town not far from here, an erythematous skin eruption similar to that of scarlet fever was a marked feature. Herpes labialis was of common occurrence, and in a few cases urticaria developed as convalescence approached. Catarrh of the stomach and bowels occasionally took the place of coryza and bronchial catarrh. In one such case observed by Dr. Wm. H. Draper the symptoms were such that typhoid fever was at first suspected; catarrhal symptoms were sometimes entirely absent. In children, swellings over the epiphyses resembling rheumatism were noted in a few cases.

Relapses were frequent, sometimes as a result of imprudence, often not. The relapse was in many cases worse than the original attack; convalescence was very slow.

Complications and sequelæ were not uncommon, and generally speaking, formed the only grave features of the disease. They usually appeared after the acute symptoms had subsided. Otitis media was perhaps the most frequent. Next to this in frequency, and the most serious of all, were those affecting the respiratory organs. There were many cases of broncho-pneumonia, pleurisy and pleuro-pneumonia. The pleurisy element was particularly marked.

A general bronchitis of the larger tubes and a localized capillary bronchitis was an unusual condition noticed in a number of cases. Dr. Loomis observed in several such cases a localized pleurisy, corresponding to the area of capillary bronchitis, while there were no signs of consolidation of lung.

Marked nervous disorders appeared in some instances. The transient neuralgia of the ordinary case became localized and persistent, or it developed into a neuritis with paralysis and absence of knee-jerk. In other cases the usual mental depression became a mild melancholia with suicidal tendencies. Such cases were observed by Dr. J. P. Kinnicutt and Dr. M. Allen Starr, the neurologist.

Adults suffered more than children, though the latter were not entirely exempt. The weak were more liable to the disease than the strong. And yet it is a singular fact that of fifty consumptives in the sanitarium at Saranac Lake not one was attacked. A diligent search for the bacillus has been made in the different laboratories but without success.

Influenza has been regarded as the precursor of cholera, and much interest will center on this point as summer approaches.

The treatment of typhoid fever was the subject at the Academy meeting of January 16th. Many of those taking part were men of large hospital experience, and teachers of medicine. Their remarks reveal that the expectant plan is that which still prevails here.

Prof. Wm. H. Thomson of the University read the paper of the evening. His efforts in treating the disease were principally confined to feeding and to intestinal antisepsis. The atrophy of the gastro-intestinal mucous membrane and the condition of the glands rendered the first difficult. There was a reversion of the gastric powers to those of an infant, and therefore he gave milk well diluted with lime water. In addition he gave ten grain doses of saccharated pepsin, with diluted hydrochloric acid every three hours. Intestinal antisepsis was accomplished by subcarbonate of bismuth, ten grains every three hours.

He regarded medicinal antipyretics as dangerous, from their mischievous action on the heart. He had used cold ever since Liebermeister had advocated it in 1871. All patients could not bear it however, no matter how mildly applied, owing to the production of prolonged vascular debility.

Dr. T. E. Satterthwaite had adopted his present plan of treatment, after trying many during an epidemic. During the first few days, he gave calomel in small doses. In the second week he began the use of hydrochloric acid, as an intestinal disinfectant. To this he added cinchona as a tonic during the third week, and toward the fourth week, two ounces of a light wine occasionally as a stimulant. Relapses were a real recurrence of the disease and not due to errors in diet. Alimentation was the backbone of the treatment, and he put his dependence on milk with lime water. It was better to under than to over-feed, medicate and stimulate. Medicinal antipyretics were dangerous. Much of the fever was due to sepsis, and hydrochloric acid often kept it in abeyance.

Prof. E. G. Janeway of Bellevue Hospital Medical College did not believe that there was any abortive treatment. In two institutions for children, where he had observed epidemics, fifty per cent. aborted without any medicinal treatment. The early use of calomel while not objectionable, was not an abortive measure. Medicinal antipyretics had replaced cold baths and packs in his practice, and they added much to the comfort of the patient by allaying certain nervous symptoms.

As stimulants he had been pleased with caffeine, digitalis and camphor in conjunction with alcohol; camphor was especially serviceable as it was an antiseptic also.

Relapses were often due to the too early use of solid food. His rule was to allow no solid food before the tenth day of absolute freedom from fever.

Dr. G. L. Peabody of New York Hospital added beef tea and broths to the usual milk diet. Late in the disease, he peptonized the milk, the sense of taste being then so modified that the patient made no objection to taking it. He did not think that relapses were often due to the early administration of solid food. He had allowed the desires of intelligent patients to guide him in many instances, and had found that a tender steak or a chop well divided could be given safely before the fever terminated. Turpentine was useful when there were meteorism and a dry tongue, but he had occasionally seen acute nephritis result from the remedy. He used medicinal antipyretics, but preferred cold baths and packs. His favorite stimulant was native wine, from two ounces to a pint daily.

Dr. J. N. Roosevelt, of Roosevelt Hospital, agreed with Dr. Peabody regarding the early administration of solid food.

Dr. Carpenter, of New Jersey, had observed excellent results from the use of nitrate of silver every three hours for intestinal hemorrhage.

Dr. Jacobi remarked that camphor could be given subcutaneously in almond oil. He approved of the use of calomel at the beginning of the disease.

Dr. A. H. Smith said that a little coffee covered the taste of peptonized milk. He thought that too much food was usually given. He used the cold coil on the abdomen to reduce temperature.

Dr. S. Baruch expressed surprise that only one of the speakers had spoken in favor of the cold bath. This could only be because the others had not used it properly. Whenever the temperature rose above 103° a bath at 65° should be given for fifteen minutes every three hours. Statistics of 30,000 cases, collected in different parts of the world where this plan had been thoroughly tested, showed a mortality rate of only two per cent.

Prof. Thomson added in conclusion that the statistics in favor of the use of cold would not justify us in ignoring it.

A few years ago the cold bath treatment was very generally employed in the hospitals here. It has now been almost entirely discarded. The reason of this is probably because it was overdone. Patients were sprinkled with ice-water or were put into much too cold water. The statistics regarding its use in the manner referred to by Dr. Baruch are, however, so important that a reaction in its favor may be expected. At the Red Cross Hospital in Lyons, France, the death rate from typhoid fever during the past twenty years was reduced from 26.2 per cent. to 16.5 per cent., by the use of occasional cold baths and antipyretic drugs. It was still further reduced to 7.3 per cent. by a much more constant use of baths. Liebermeister states that at Basle a reduction from 26 per cent. to 16.2 per cent., and eventually to 8.8 per cent., took place under the thorough use of cold baths. In the German Military Hospitals the mortality rate from typhoid fever was in a similar manner reduced 25.8 per cent. to 15 per cent. and then to 8.9 per cent.

